# MicroRNAs in Breast Cancer Bone Metastasis Formation and Progression: An Overview on Recent Progress in This Research Field

**DOI:** 10.3390/ncrna11060080

**Published:** 2025-12-18

**Authors:** Margherita Puppo

**Affiliations:** IRCCS Ospedale Policlinico San Martino, Largo Rosanna Benzi 10, 16132 Genova, Italy; margherita.puppo@hsanmartino.it

**Keywords:** breast cancer, bone metastasis, microRNA, miRNA, miR, ncRNA, biomarkers, target therapy, osteoclast, osteoblast

## Abstract

Bone metastasis is a common and severe complication in advanced stages of breast cancer (BC) that is characterised by limited treatment options and poor patient prognosis. MicroRNAs (miRNAs) are a large class of regulatory small non-coding RNAs (ncRNAs) expressed by cells. Moreover, miRNAs can be released by cells into the blood and lymphatic streams, acting as distant cell-to-cell communicators. Of note, miRNAs have pivotal roles in the metastatic progression of BC to bone. This review summarises the most recent findings on miRNAs and their mRNA targets in driving BC bone metastasis. Furthermore, the potential clinical uses of miRNAs as future therapeutic targets/agents or biomarkers for BC bone metastasis are discussed.

## 1. Introduction

Breast cancer (BC) is the second most common cancer in the world [1] and the most prevalent among the female population [2]. It has been predicted that by 2050, there might be 3.2 million new breast cancer cases and 1.1 million breast cancer-related deaths each year worldwide [2]. Even if most cases of BC account for localised or locally invasive (adjacent structures or regional lymph nodes) events, and less than 6% of cases have distant metastasis at the time of the diagnosis [3], BC is ranked as the fourth leading cause of cancer-related mortality [4]. BC mortality is usually related to advanced stages of the disease, as BC progresses to distant organs, with bone being the most common site for BC metastases [4]. BC metastasis occurs when tumour cells break away from their primary site, invading surrounding tissues, and then intravasate into blood or lymphatic vessels, circulate, extravasate from circulation, and finally reach distant sites where they form micro-metastasis of few cells that may eventually evolve in detectable metastases [5]. In European countries, both screening tests (i.e., mammography, ultrasounds, magnetic resonance imaging, physical examination) and advanced therapies (i.e., chemotherapy, immunotherapy, targeted therapy, surgical interventions, radiation therapy, endocrine therapy, antibody-drug conjugate) have greatly increased the five-year overall survival rate for patients with an early-stage of BC [6]. Conversely, metastatic BC has a poor survival rate, with a median overall survival of three years, with some variations based on BC subtype, patient characteristics, and access to treatment [7]. Moreover, in case of BC bone metastasis, patients’ quality of life is further negatively impacted due to the presence of skeletal-related events such as pathologic fractures, spinal cord compression, bone radiation therapy, and bone surgery [8]. In these patients, both local and systemic treatments are required to manage bone metastases, with bone turnover modulators reducing the risk of skeletal complications and improving pain management [8,9]. Despite significant advantages in the early diagnosis and treatment of BC over recent decades, more is needed to predict the risk of developing BC and to reduce suffering and mortality from metastatic BC.

MicroRNAs (miRNAs, miRs), a class of small non-coding RNA (ncRNA) molecules, are often dysregulated in cancer. For this reason, miRNAs hold great potential as future biomarkers and/or targeted therapy in pre-clinical settings for BC [10,11,12], as well as other cancers. In cells, miRNAs post-transcriptionally regulate gene expression by binding to complementary target messenger RNAs (mRNAs), leading to mRNA translational inhibition or degradation [10,13]. However, the molecular mechanisms by which miRNAs drive cancer formation and progression are complex, as miRNAs can affect multiple targets at the same time [10,14,15]. Moreover, miRNAs can be secreted by donor cells and modulate the gene expression of recipient (and even distant) cells. The ability of miRNAs to act as distant modulators markedly complicates the level of complexity of molecular mechanisms that contribute to tumorigenesis and metastasis. In fact, a single miRNA can be able to modulate both cancer cells and surrounding cells, including organ-specific and immune system cells [10,16,17].

This review aims to present recent findings on miRNAs as biomarkers and targeted therapies in BC bone metastasis, summarising current research on miRNA’s multiple roles in the regulation of BC in bone.

## 2. Breast Cancer Bone Metastasis

Breast cancer (BC) often metastasises to bone [4,5], with oestrogen receptor (ER)-positive BC exhibiting a particular propensity for this metastatic site [18]. In general, the bone provides a fertile soil for disseminating cancer cells (DTCs), since the availability of numerous growth factors and cytokines that are produced by bone marrow resident cells (i.e., haematopoietic stem cells, mesenchymal stem cells, endothelial cells, osteoblasts, osteoclasts) [4,19]. Moreover, bone marrow is a hypoxic environment, which usually promotes tumour progression and enhances the metastatic potential of cancer cells [19]. Finally, in bone marrow, it also exists two highly specialised niches—the endosteal “osteoblastic” niche and the perivascular niche—that provide an ideal microenvironment for metastasis, allowing DTCs to co-opt these physiological niches in order to promote their own survival and outgrowth [19,20].

Besides the existence of a particularly favourable soil for BC cells in bone, it is now widely accepted that primary BC cells, even at an early stage, can secrete a variety of factors that allow distant sites to be ‘prepared’ to host disseminating cancer cells: a process known as the pre-metastatic niche (PMN) formation [4,5,21,22]. Osteoclasts and osteoblasts, two key resident cell types in bone, play crucial roles in the PMN formation, since they can respond to the secreted factors from BC cells. These factors can induce alterations in their maturation as fully functional cells and/or in their activity on bone remodelling [4,5,20,21,22].

Osteoclasts are monocyte-derived, multinucleated cells that degrade the bone matrix (bone resorption) [4]. Their differentiation, also known as osteoclastogenesis, is promoted by several factors, including the receptor activator of nuclear factor kappa-beta ligand (RANKL), the macrophage colony stimulating factor (M-CSF), and various cytokines that are released by osteoblasts or other bone resident cells in physiological conditions [4]. Osteoblasts, which derive from bone marrow mesenchymal stem cells, contribute to the production of the bone extracellular matrix that subsequently mineralises (bone formation) [4]. Their differentiation is sustained by local factors, such as the transforming growth factor beta (TGF-β), bone morphogenic proteins (BMPs), and the activation of the Wingless-INT (Wnt) pathway [4].

While being in the early stages of metastasis, BC cells can start altering the fine-tuned bone homeostasis with the production of soluble factors, favouring the formation of the PMN; at a later stage, the activities of already disseminated BC cells (BC-DTCs) can result in a complete disequilibrium between osteoclast/osteoblast activities. Generally, it has been described that BC-DTCs can take advantage of an increased osteoclastic activity, which is typically characterised by an increase in growth factors released from the resorbed bone matrix that sustain BC cell proliferation [4,5,20]. However, it is normally difficult to evaluate the starting point of this ‘vicious cycle’, and clinically, we usually only detect its presence in the late stages of the metastatic progression, when bone-related skeletal events occur.

## 3. Research Strategy for This Review

This comprehensive literature review has been mainly realised by using the PubMed^®^ database that comprises more than 39 million citations for the biomedical literature from MEDLINE, life science journals, and online books. The following search string was applied: (“miRNA” OR “microRNA” OR “miR-”) AND (“breast cancer”) AND (“bone metastasis” OR “metastatic” OR “bone” OR “secondary tumours”). Only original articles—excluding preprints—published in English from 1 January 2023 to 1 September 2025 have been considered, for a total of 181 results. Then, all articles have been screened for eligibility, focusing on studies that discuss the chosen topic for this review. Here, the selected articles are presented in ascending order, based on the miRNA described in the study (Table 1).

## 4. MiRNAs in BC Bone Metastasis

MiRNAs, discovered by Nobel laureates Dr Victor Ambros and Dr Gary Ruvkun [23], are a large class of short (18–22 nucleotides) non-coding RNAs (ncRNAs) that regulate gene expression within cells by either inhibiting protein translation or promoting the degradation of messenger RNA (mRNA) targets [10,13,24]. Unlike other ncRNAs, the biogenesis of miRNAs is a highly regulated process that requires the activity of several enzymes, such as Drosha and Dicer, on miRNA precursors in order to obtain a mature, functional form that complexes with Argonaute proteins to form an effector complex called the RNA-induced silencing complex (RISC) [25]. This complex uses the miRNA sequence as a guide to recognise complementary sequences on mRNA targets, leading to their translational repression [10,13,24]. This post-transcriptional regulation operated by miRNAs is of crucial importance to maintain physiological cellular functions. Thus, even small changes in the expression levels of miRNAs can result in substantial changes in mRNA expressions and cell behaviour, as seen in cancer cells [10,13,24].

**Figure 1:**
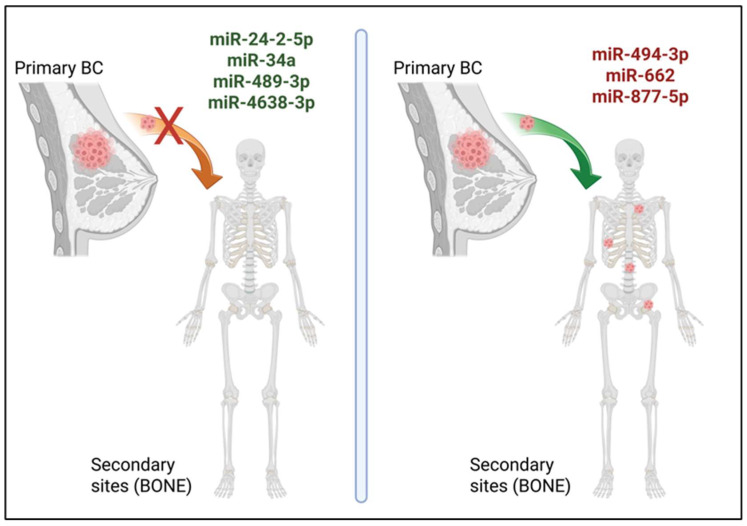
Metastatic BC progression to bone regulated by miRNAs. In recent studies, miR-24-2-5p, miR-34a, miR-489-3p, and miR-4638-3p (in green, left panel) are shown to act as oncosuppressor-miRs in BC bone metastasis, while miR-494-3p, miR-662, and miR-877-5p (in red, right panel) have been described as onco-miRs in this process. Created with the image and illustration software ‘BioRender ‘(https://app.biorender.com/, accessed on 25 November 2025).

In cancer, miRNAs are often classified as onco-miRs or tumour suppressor miRNAs (oncosuppressor-miRs). Onco-miRs are typically upregulated in cancer cells and can repress oncosuppressor genes, thereby promoting tumorigenesis. Conversely, oncosuppressor-miRs are usually downregulated in cancer cells as they target oncogenes and inhibit tumour progression [10,24]. Of note, the expression levels of onco-/oncosuppressor-miRs, or even their roles, may change with time or based on the type of cancer type [15]. It is not surprising that some miRNAs can regulate and promote a specific step of the tumour progression, sometimes making the understanding of the consequences of miRNA dysregulation in cancer challenging. Many miRNAs have been reported to be involved in BC progression to bone by regulating every step from the acquisition of a more invasive and aggressive phenotype by BC cells to their dissemination, seeding, and proliferation in bone [5,10,24,26,27]. Here, recent findings on miRNA roles in BC progression to bone will be presented and further discussed (Figure 1).

### 4.1. MiR-24-2-5p

MiR-24-2-5p is one of the members of the well-known miR-23~27~24 cluster. This cluster was first described in 2010, when its functional role in regulating B cell development was demonstrated [28]. Since then, all members have been extensively investigated for their role in several diseases, including cancer [29,30,31]. In the human genome, miR-23~27~24 consists of two sub-clusters, miR-23b~27b~24-1 and miR-23a~27a~24-2, on chromosomes 9 and 19, respectively. Even if they share a common site of origin, the expression and functional activity of each individual miRNA are independent from the other members of the cluster, with observed differences in the levels of miR-23, miR-27, and miR-24 across different cell types [31]. In BC, it has been shown that the miR-24-2 precursor (pre-miR-24-2) is associated with a decrease in BC tumorigenesis, and that miR-24-2-5p (a mature form) is directly responsible for repressing PKC-alpha levels, which is important for BC cell survival and drug resistance [32]. Recently, miR-24-2-5p has been further investigated in the progression of BC to bone and its role in the bone micro-environment [33]. In this study, low circulating miR-24-2-5p levels have been associated with a higher risk of developing bone metastases in early-stage ER-positive BC patients. Moreover, the overexpression of miR-24-2-5p in two BC cell models, MDA-MB-231 (triple-negative, TNBC) and MCF7 (ER-positive), reduced their malignant traits such as migration, invasion, proliferation in vitro, and decreased bone metastasis in vivo, indicating a protective role of this miRNA during invasive BC cell progression to bone. Additionally, BC cell-derived miR-24-2-5p was able to reduce osteoclast differentiation in vitro and bone resorption in vivo, adding a complementary level of regulation of this miRNA occurring during BC bone metastasis formation. Finally, lower levels of endogenous miR-24-2-5p were detected in mature murine osteoclasts compared to their precursor cells, further suggesting an inhibitory role for miR-24-2-5p during murine osteoclastogenesis [33]. Previous studies have also implicated miR-24-2-5p in bone homeostasis, demonstrating that it reduces Gnai3 expression levels to ultimately suppress osteogenic differentiation [34]. Overall, miR-24-2-5p appears to be a promising candidate for translational research in BC bone metastasis, given its capacity to modulate multiple yet complementary pathways on both BC cells and bone-resident cells, such as osteoclasts and osteoblasts. This ‘dual’ role within the bone-microenvironment of miR-24-2-5p, taken as an example, should prompt us to evaluate the effect of a specific delivery system for miR-24-2-5p mimics in carriers—such as artificial liposomes, nanoparticles, or viruses—in animal models of bone metastasis, with the ultimate goal being to suggest new preventive treatments for early-stage BC patients at high risk of developing bone metastases.

### 4.2. MiR-34a

MiR-34a is the most abundant member of the miR-34 family, which also comprises miR-34b and miR-34c, and one of the most studied miRNAs with the role of oncosuppressor-miR in cancer, including BC [35]. Three anti-tumour mechanisms have been described for miR-34a: (1) induction of G0/G1 arrest and consequent decrease in tumour cell proliferation; (2) downregulation of epithelial-to-mesenchymal (EMT) processes and suppression of tumour cell motility; and (3) inhibition of cancer cell autophagy and induction of apoptosis [35]. The miR-34a mimic (MRX34) incapsulated within liposome-like nanoparticles (NOV40) was the first miRNA mimic to be evaluated in a phase I study in patients with advanced solid tumours [36]. Although this trial was prematurely closed due to immune-mediated adverse events in some participants [36], it provided a fundamental proof-of-concept for future miRNA-based cancer therapies. To address concerns regarding stability, non-specific delivery, and toxicity, a modified version of miR-34a conjugated with folate (FM-FolamiR-34a) was developed and tested in animal models of BC [37]. Successful results in inhibiting tumour growth were obtained in some mice [37], suggesting that a new class of miRNA-based molecules with anti-tumoral activity could be produced for future therapeutic approaches. In the context of BC bone metastasis, the potential application of miR-34a mimicking anti-cancer gene therapy needs to take into consideration the specificity of these molecules to bone as well as their uptake by bone resident cells. In a recent study, a bone-targeted delivery system for miR-34a for gene therapy specifically for bone-associated metastatic BC has been developed in a preclinical setting [38]. In this approach, miR-34a was loaded into a non-viral gene vector (PCA/miR-34a), which was designed to prevent its degradation during blood circulation and to enhance delivery and distribution specifically to bone, with the final aim being to promote BC cell apoptosis and reduce bone tissue erosion [38]. The anti-tumour efficacy of PCA/miR-34a was tested in murine models of BC bone metastasis following a tail vein injection of the nanoparticle solution. Interestingly, the volumetric statistical analysis of the BC-derived tumour in the leg of mice treated with PCA/miR-34a showed a lower tumour growth compared to controls, and a minor incidence of secondary tumours in lungs was also reported [39]. The protein expression of Bcl-2, a traditional anti-apoptotic factor in progressive tumour cells that can be down-regulated by miR-34a, was found to be significantly reduced in tumour tissues of mice treated with PCA/miR-34a compared to controls, where tumour cells also showed reduced proliferation and increased apoptosis [39]. Overall, the use of bone-targeted gene delivery vectors carrying tumour-suppressive miRNAs like miR-34a represents a promising therapeutic strategy for BC bone metastasis, warranting further evaluation in clinical trials.

### 4.3. MiR-130a

MiR-130a originates from a gene located on chromosome 11, and its mature sequence differs from that of miR-130b, whose gene is located on chromosome 22 instead. The sequences of miR-130a and miR-130b differ by only two nucleotides at positions 11 and 13; however, their functional role and targets can be completely different [39]. In cancer, the expression of miR-130a has been found to be aberrant in several types of cancer, including BC, and miR-130a could act as an onco- or oncosuppressor-miR depending on the biological context [40]. For example, the overexpression of miR-130a inhibited BC cell proliferation, invasion, and migration by directly targeting the 3′UTR of RAB5A mRNA [41]. However, miR-130a expression levels related to cancer recurrence and its detection in liquid biopsies remain contradictory in the literature [42]. In a recent study, the expression levels of oestrogen receptor 1 (ESR-1), long ncRNA HOTAIR, and miR-130a were evaluated for their ability to predict BC stage and metastasis in a cohort of 45 patients with primary BC who did not receive neoadjuvant chemo- or radiotherapy before surgery [43]. Circulating miR-130a expression levels were more elevated in BC patients compared to healthy controls, with luminal B BC patients having the highest expression levels of miR-130a compared to patients with other BC sub-types [43]. Interestingly, miR-130a expression levels were higher in stage-I compared to stage-IV BC patients, suggesting a correlation between miR-130a expression levels and disease progression [43], which is still unclear in the case of bone metastasis. Further studies are indeed necessary to establish the association between circulating miR-130a expression levels and metastatic progression of BC, particularly in bone.

### 4.4. MiR-223-3p

MiR-223-3p, with miR-223-5p, is transcribed from the *MIR223* gene located on chromosome Xq12, and it was first described for its role in the modulation of haematopoietic lineage differentiation [44,45]. Then, it was demonstrated that miR-223-3p was also involved in regulating human embryonic stem cells, osteoclast differentiation, immune cell differentiation and activation, and crucially, it was involved in cancer development [45]. The role of miR-223-3p in cancer appears to be cancer-dependent and influenced by the presence of its targets in cells: for this reason, both oncosuppressive and oncogenic roles have been described for miR-223-3p [45]. In BC, miR-223-3p may suppress BC development by targeting oncogenic transcripts, including epithelial cell transforming 2 (ECT2), Profilin 2 (PFN2) and NOD-, LRR- and pyrin domain-containing protein 3 (NLRP3) as examples, or act as a onco-miR by enhancing cell proliferation, migration, invasion, and EMT through Hippo/Yap1or Notch signalling pathways as examples [45]. There is evidence that miR-223-3p might promote BC metastasis by regulating the lipid metabolism, which dysregulation is linked to tumour progression, by directly targeting SCARB1 (thus, suppressing cholesterol intake) and HMGCS1 (thus, suppressing cholesterol biosynthesis) [46]. Recently, the regulatory role of miR-223-3p on lungs and BC cell growth in bone has been described [33]. This study mainly focusses on the effect of Ugonin P, a flavonoid from *Helminthostachys zeylanica* Hook, previously known for its antioxidant and anti-cancer effects. The researchers investigated Ugonin P’s impact on lung- and BC-promoted osteoclast differentiation and bone metastasis progression. Interestingly, Ugonin P reduced MDK production (a heparin-binding protein that promotes cell proliferation, survival, migration, and EMT) through the upregulation of miR-223-3p expression levels. In fact, Ugonin P’s treatment of BC cells was able to increase miR-223-3p expression in a dose-dependent manner, and a forced repression of miR-223-3p expression levels was able to block the suppressive effects of Ugonin P on MDK at both mRNA and protein levels in vitro [47]. The effects of the modulation of miR-223-3p in BC cells on bone metastasis should be further investigated with a dedicated experimental design in vitro and further validation in animal models of bone metastasis.

### 4.5. MiR-489-3p

MiR-489-3p has been described to mainly act as an oncosuppressor-miR in various cancers. As examples, miR-489-3p has been shown to inhibit cell proliferation, migration, and invasion in glioblastoma [48], cell proliferation and metastasis in pancreatic cancer [49], and cell proliferation and migration in bladder cancer [50], and to enhance ferroptosis in gastric cancer [51]. A recent study investigated the role of the tumour-derived exosomal long ncRNA MIR193BHG on its contribution to BC bone metastasis through the negative regulation of the miR-489-3p/DNA methyltransferase 3A (DNMT3A) signalling axis [52]. In this study, the mRNA and protein levels of DNMT3A, known to regulate osteoclast differentiation, were significantly reduced in bone marrow macrophages treated with BC-derived exosomes, where lncRNA-MIR193BHG was silenced in their relative cells of origin [52]. Interestingly, both DNMT3A and lncRNA-MIR193BHG were found to be direct targets of miR-489-3p, suggesting that lncRNA-MIR193BHG may promote osteoclast differentiation and function by competitively binding to miR-489-3p, thereby preventing miR-489-3-dependent DNMT3A downregulation [52]. This study nicely highlights how different classes of ncRNAs can be involved in the same regulatory pathways that promote BC’s progression to bone.

### 4.6. MiR-494-3p

MiR-494-3p is primarily recognised as an onco-miR in various cancer types. For instance, it has been shown to enhance an aggressive phenotype in lung cancer cells [53,54], promote the progression of endometrial cancer [55], facilitate the progression of bladder cancer [56], and its inhibition in exosomes decreases gastric cancer cell proliferation [57]. A role of miR-494-3p in BC has been demonstrated in a few studies. In fact, miR-494-3p was shown to directly target TRIM21, an E3 ubiquitin ligase, that served as tumour suppressor during BC progression [58]. Moreover, a regulative mechanism occurring in BC has been described, in which MEG3 was able to downregulate miR-494-3p expression levels, while miR-494-3p was shown to target the DNA methyltransferase 1 (DNMT1) in a cell model of BC. In this study, both MEG3 upregulation and miR-494-3p downregulation were able to inhibit the malignant behaviour of BC cells in vitro [59]. On the other hand, a study has demonstrated that the self-renewal activity of BC cells in mammospheres treated with hinokitiol (β-thujaplicin), a tropolone-related compound with anti-microbe, anti-inflammation, and anti-tumour effects, was reduced, despite an increase in miR-494-3p expression levels [60]. Here, the authors suggested that miR-494-3p in BC cells with stem-like properties (BCSCs) could directly target BMI1 mRNA—a gene known to positively regulate the self-renewal capability of BCSCs—thereby inhibiting mammospheres-forming capability and reducing BCSCs’ tumorigenicity [60], thus suggesting a oncosuppressive role for miR-494-3p. In BC bone metastasis, miR-494-3p, together with miR-4508 and miR-6869-5p, was identified as an osteoclastogenic miRNA that is present in exosomes secreted by RAS-activated BC cells [61]. In general, RAS activation is a key determinant of BC progression and metastasis [61]. Interestingly, the forced downregulation of miR-494-3p expression levels in BC cells was able to abolish exosome-mediated, RANKL-induced osteoclastogenesis. Conversely, miR-494-3p overexpression in bone marrow-derived macrophages enhanced RANKL-induced osteoclastogenesis by targeting LGR4 and SEMA3A in the bone microenvironment [61]. Notably, treatment with a miR-494-3p inhibitor significantly suppressed the exosome-mediated promotion of osteolytic bone lesions in a murine model of BC bone metastasis [61], suggesting the critical role of miR-494-3p in osteolytic bone metastasis processes. These findings deserved further investigation into miR-494-3p as a potential therapeutic target.

### 4.7. MiR-662

The overexpression of miR-662, together with miR-192-5p and miR-192-3p, was first described to be associated with a high risk of developing distant metastases in early-stage squamous cell lung cancer (SCC) [62]. Subsequently, miR-662 was found to enhance clonogenicity and motility, mediate resistance to etoposide (but not cisplatin), and promote WNT-pathway gene expression in SCC cells [63]. More recently, miR-662 has been associated with the progression of BC to bone [64]. In particular, high expression levels of serum-circulating miR-662 were associated with a higher risk of developing bone metastasis in early-stage, ER-positive BC patients [64]. While miR-662 expression was not found to be associated with a particular BC sub-type, its forced expression in a human TNBC cell model was able to increase the proliferative, migratory, and invasive abilities of BC cells. When tested in vivo, miR-662 overexpression in BC cells was shown to promote the formation of osteolytic bone metastasis, even if a period of latency was observed in an early phase of BC metastatic growth [64]. This latency, often noted clinically in ER+ BC patients, could be explained by the secondary effect of miR-662 on osteoclasts. Specifically, miR-662 reduced osteoclast differentiation from their precursor cells in the presence of the conditioned medium from miR-662-overexpressing BC cells in vitro [64]. High-throughput analyses revealed that miR-662 decreases global protein synthesis, which is associated with a cancer cell stemness. This suggests that miR-662 contributes to the acquisition of a stem-like phenotype of BC cells, which was further demonstrated by the increase in stem-related genes (NOTCH1, WNT7b, ZEB1, TCF3, and SNAI2) and ALDH enzymatic activity upon miR-662 overexpression in BC cells [64]. Even if miR-662-driven molecular mechanisms that are responsible for the decrease in global protein synthesis need to be clarified, miR-662 is a promising target for gene therapy, as well as a potential biomarker for BC bone metastasis.

### 4.8. MiR-877-5p

MiR-877-5p has been described in various cancers, including gastric [65,66,67], prostate [68,69], cervical [70,71], lung [72,73], and breast [74,75] cancers; however, its role as onco- or oncosuppressor-miR seems to be related to the type and/or stage of the relative cancer. Recently, a novel molecular pathway involving the long ncRNA TRG-AS1, miR-877-5p, and WISP2 has been shown to regulate BC progression to bone [76]. Low expression levels of TRG-AS1 were associated with longer disease-free survival for BC patients. Moreover, TRG-AS1 was downregulated in both primary tissues and, even at a lower expression, in the bone metastasis tissues of BC patients [76]. Interestingly, TRG-AS1 and WISP2 were identified to be direct targets of miR-877-5p. The overexpression of miR-877–5p, as the inhibition of TRG-AS1, was able to significantly increase the proliferative and invasive abilities of bone-tropic BC cells, increase BC-induced osteoclastogenesis, and decrease osteoblastic differentiation in vitro [76]. On the other hand, WISP2 silencing was able to rescue the effects of TRG-AS1 on osteoclastic and osteoblastic differentiations [76], suggesting a regulative pathway involving these two molecules and miR-877-5p. Although animal studies were conducted to evaluate the effect of TRG-AS1 knockdown on BC bone metastases [76], a dedicated experimental design in vivo for miR-877-5p was not performed. Since its activity on bone resident cells was proven [76], additional and dedicated studies should be conducted to further investigate the role of miR-877-5p in BC bone metastasis.

### 4.9. MiR-4638-3p

The current literature on the role of miR-4638-3p in cancer is limited to two studies conducted by the same research group. In the first study, the authors investigated miR-4638-3p’s role during the TGF-β1-induced EMT in BC cells [77]; in the second study, they validated the functional role of miR-4638-3p in the metastatic progression of BC to bone [78]. In more detail, it was demonstrated that TGF-β1 downregulated miR-4638-3p expression in a BC cell model [77]. Moreover, the forced expression of miR-4638-3p was able to reduce ATF-3 expression, resulting in the downstream regulation of Runx2 and MMP-13, which reduced cell proliferation, invasion, and migration, and induced G0/G1 cell cycle arrest and apoptosis [77]. ATF-3 and MMP-13 mRNAs were also found to be directly regulated by miR-4638-3p [77]. Interestingly, mice injected in the caudal tail artery with miR-4638-3p-overexpressing BC cells showed a decreased expression of bone resorption marker genes and a reduction in BC-induced osteolytic lesions [78]. Overall, mice injected with miR-4638-3p-overexpressing BC cells showed a better micro-architecture of the trabecular network, suggesting that miR-4638-3p could reduce BC bone metastasis in vivo [78]. As for miR-24-2-5p and miR-34a, it could be interesting to evaluate the effect of the delivery of this microRNA in gene vectors in pre-clinical models and clinical trials.

### 4.10. Exosomal miRNAs

MiRNAs can circulate in biological fluids, such as blood and lymph, where they interact with core proteins that protect them from massive degradation, both as free and embedded forms [5]. As embedded forms, miRNAs are usually found in extracellular vesicles (EVs)—a heterogeneous class of vesicle organelles that includes exosomes, microvesicles and apoptotic bodies—released by cells. Understanding of the role of EVs, as well as their miRNA cargo, is fundamental to comprehend the cell-to-cell communication occurring during metastatic BC. As examples, both miR-24-2-5p (as oncosuppressor-miR) and miR-662 (as onco-miR), two miRNAs involved in BC bone metastasis progression as previously described, have been shown to be cargoes of BC cell-derived EVs, even at a higher level when those miRNAs were overexpressed in BC cells [33,64]. This suggests that changes in the miRNA content inside EVs could be the consequence of an altered expression of miRNAs in EV-producing BC cells. Thus, an altered miRNA expression in BC cells can be reflected in the altered miRNA content of EVs and finally, this results in important consequences for adjacent and/or distant cells such as bone resident cells, whose activities in bone can be altered to promote BC metastasis [5,33,64]. Lately, exosomes, EVs ranging from 30 to 150 nm in diameter and with a lipid bilayer, have attracted growing interest for their key role in cell communication and their potential as therapeutic carriers, including in BC, due to their improved bioavailability, greater stability, and reduced off-target cytotoxicity and immunogenicity [79]. Cargo molecules, including miRNAs, are selectively incorporated into exosomes through molecular sorting pathways, which are modulated by specific proteins such as CD9, CD63, and CD81 [79]. Notably, recent evidence suggests that specific exo-motifs in miRNAs may be recognised by RNA binding proteins, ultimately leading to a sequence-based miRNA sorting into exosomes [80]. For these reasons, the study of exosomal miRNAs and RNA binding proteins is quite interesting in the context of BC bone metastasis. In a recent study, the miRNA content and role of EVs from BC, in which LSD1—a BC oncosuppressor—was silenced, have been investigated [81]. Isolated exosomes from LSD1 KD BC cells were intravenously injected in murine models of bone metastasis and significantly promoted osteolytic BC metastasis [81]. Moreover, the expression of miR-6881-3p, miR-6726-3p, miR-34c-3p, and miR-4457 was decreased in LSD1 KD exosomes compared to controls [81]. Finally, LSD1 was described to control miR-6881-3p sorting into exosomes by regulating the expression of the RNA-binding protein hnRNPA2B1, with the ultimate effect being to remodel the PMN during BC bone metastasis [81]. This study also suggests that new therapeutic approaches should take in consideration the strategy of inhibiting exosome uptake by target cells to counteract pro-tumoral signals.

**Table 1:** List of recent (from 1 January 2023 to 1 September 2025 on PubMed^®^) miRNAs with a role in the progression of BC to bone. Some molecular mechanism that have not been fully addressed in the studies are indicated in this table as “n/a”.

MiRNA ID	Ref.	Date	MiRNA’s Roles in BC Bone Metastasis	Validated Direct Target/s	Clinically Related Evidence	Other Mechanisms in BC
miR-24-2-5p	[33]	2024	Oncosuppressor: Its overexpression reduces (i) migration, invasion, proliferation of BC cells in vitro, (ii) bone metastasis in vivo, and (iii) osteoclastogenesis in vitro.	n/a	Low serum-circulating levels in early-stage ER-positive BC patients with higher risk of developing bone metastasis [33].	Repression of PKC-alpha levels that decreases BC tumorigenesis [32].
miR-34a	[39]	2023	Oncosuppressor: It (i) promotes BC cell apoptosis, (ii) reduces bone tissue erosion, and (iii) reduces tumour growth in vivo.	BCL-2	MiR-34a mimic (MRX34) was evaluated in a phase I study (NCT01829971) that closed prematurely due to immune-mediated adverse events [36].	Induction of G0/G1 arrest; downregulation of EMT; inhibition of cancer cell autophagy; and induction of apoptosis [35].
miR-130a	[43]	2023	Still to be clarified.	n/a	High circulating miR-130a levels in BC patients (especially in luminal B subtype) compared to healthy controls [43].	MiR-130a overexpression inhibits BC cell proliferation, invasion, and migration by directly targeting RAB5A [41].
miR-223-3p	[47]	2025	Still to be clarified. Ugonin P reduced MDK production through the upregulation of miR-223-3p levels.	n/a	n/a	As oncosuppressor-miR, it decreases BC development by targeting ECT2, PFN2, and NLRP3. As onco-miR, it (i) enhances cell proliferation, migration, invasion, and EMT through Hippo/Yap1or Notch signalling pathways [45], and (ii) regulates lipid metabolism by targeting SCARB1 and HMGCS1 [46].
miR-489-3p	[52]	2025	Oncosuppressor: It indirectly reduces osteoclast differentiation and activity.	DNMT3A, lncRNA-MIR193BHG	n/a	n/a
miR-494-3p	[61]	2023	Tumour promoter: (i) It promotes osteoclastogenesis by being released in exosomes from RAS-activated BC cells and (ii) miR-494-3p inhibitor treatment suppresses exosome-mediated promotion of osteolytic bone lesions in vivo.	LGR4, SEMA3A	n/a	As onco-miR, it targets TRIM21 during BC progression [58]; miR-494-3p downregulation inhibits malignant behaviour of BC cells in vitro [59]. As oncosuppressor-miR, it inhibits BC cell mammospheres-forming capability and decreases tumorigenicity [60].
miR-662	[64]	2023	Tumour promoter: Its overexpression (i) enhances migration, invasion, proliferation of BC cells in vitro, and bone metastasis in vivo, and (ii) reduces osteoclastogenesis in vitro.	n/a	High serum-circulating levels in early-stage ER-positive BC patients with higher risk of developing bone metastasis [64].	n/a
miR-877-5p	[76]	2023	Tumour promoter: Its overexpression (i) increases bone-tropic BC cell proliferation and invasion, (ii) increases BC-induced osteoclastogenesis, and (iii) decreases osteoblastic differentiation in vitro	TRG-AS1, WISP2	n/a	It is a potential link between TNBC development and metabolic syndrome [74]; it inhibits EMT by targeting FGB [75].
miR-4638-3p	[78]	2024	Oncosuppressor: Its overexpression reduced BC-induced osteolytic lesions in vivo.	ATF-3, MMP-13	n/a	MiR-4638-3p overexpression reduces ATF-3 expression, resulting in reduced BC cell proliferation, invasion, and migration, and induced G0/G1 cell cycle arrest and apoptosis [77].
Exosomal miRs: miR-6881-3p, miR-6726-3p, miR-34c-3p, miR-4457	[81]	2024	Oncosuppressors: Their expressions were decreased in LSD1 KD exosomes.	n/a	n/a	EV-miRNAs can be onco- or oncosuppressor-miRs in BC [33,64].

## 5. Conclusions and Future Perspectives 

MiRNAs are critical regulators of the metastatic progression of BC cells to bone. The recent literature highlights several key roles of these small molecules in both BC and bone resident cells (Table 1), making it evident that future studies need to evaluate the effect of miRNAs across various cell types—such as cancer, bone resident, and immune cells—as well as their role as important long-distance cell-to-cell communicators. Even if some miRNAs have been recently described to play a role as onco-miRs (e.g., miR-494-3p, miR-662, miR-877-5p) or oncosuppressor-miRs (e.g., miR-24-2-5p, miR-34, miR-4638-3p), and the modulation of their expression levels has been successfully achieved in pre-clinical settings in vitro and in vivo, their future use as therapeutic targets or agents urgently required further investigations. Surely, miRNA-based therapies are very promising, and they hold the potential to radically change the clinical approach to cancer treatment. However, possible off-target effects need to be carefully evaluated alongside the design of efficient delivery vectors for miRNA-mimics or -inhibitors. More translational studies should be conducted with the most promising miRNA candidates, as well as new early-phase clinical trials. The common mechanisms of action for miRNAs in bone metastasis from different cancers—such as prostate and lung cancers that have a high propensity to migrate to bone—should be evaluated as well. Another use of miRNAs in BC patients could be as biomarkers, particularly in liquid biopsies that are minimally invasive procedures compared to tissue biopsies. In the future, the integration of a panel of circulating miRNAs in addition to existing biomarkers might be used to evaluate the risk of BC progression, the presence of micro-metastasis whose presence cannot be detected by current methods, and to monitor treatments. This might be extended to other type of cancers besides BC. In conclusion, miRNA research should be encouraged at pre-clinical and clinical levels, in both early and advanced BC stages, as well as in other bone-tropic cancers in order to achieve clinical advancements for the treatment and diagnosis of bone metastasis.

## Data Availability

Not applicable. No new data were generated.

## Abbreviations

The following abbreviations are used in this manuscript:

BC	Breast Cancer
BCSC	Breast Cancer Stem Cell
BMP	Bone Morphogenic Protein
DTC	Disseminating Tumour Cell
EMT	Epithelial-to-Mesenchymal Transition
ER	Oestrogen Receptor
EV	Extracellular Vesicles
M-CSF	Macrophage Colony Stimulating Factor
miRNA, miR	MicroRNA
mRNA	Messenger RNA
PMN	Pre-Metastatic Niche
RANKL	Receptor Activator of Nuclear Factor Kappa-Beta Ligand
RISC	RNA-Induced Silencing Complex
SCC	Squamous Cell Lung Cancer
TGF-β	Transforming Growth Factor Beta
TNBC	Triple-Negative Breast Cancer
WNT	Wingless-INT
